# Supplementary material for: Identification, Quantification, and Characterization of HIV-1 Reservoirs in the Human Brain
Source: Cells. 2022 Aug 2;11(15):2379. doi: 10.3390/cells11152379 (PMC9367788; doi:10.3390/cells11152379)
Supplement: Supplementary file 1 [file cells-11-02379-s001.zip › cells-1820742-supplementary.pdf]

Supplementary Materials:

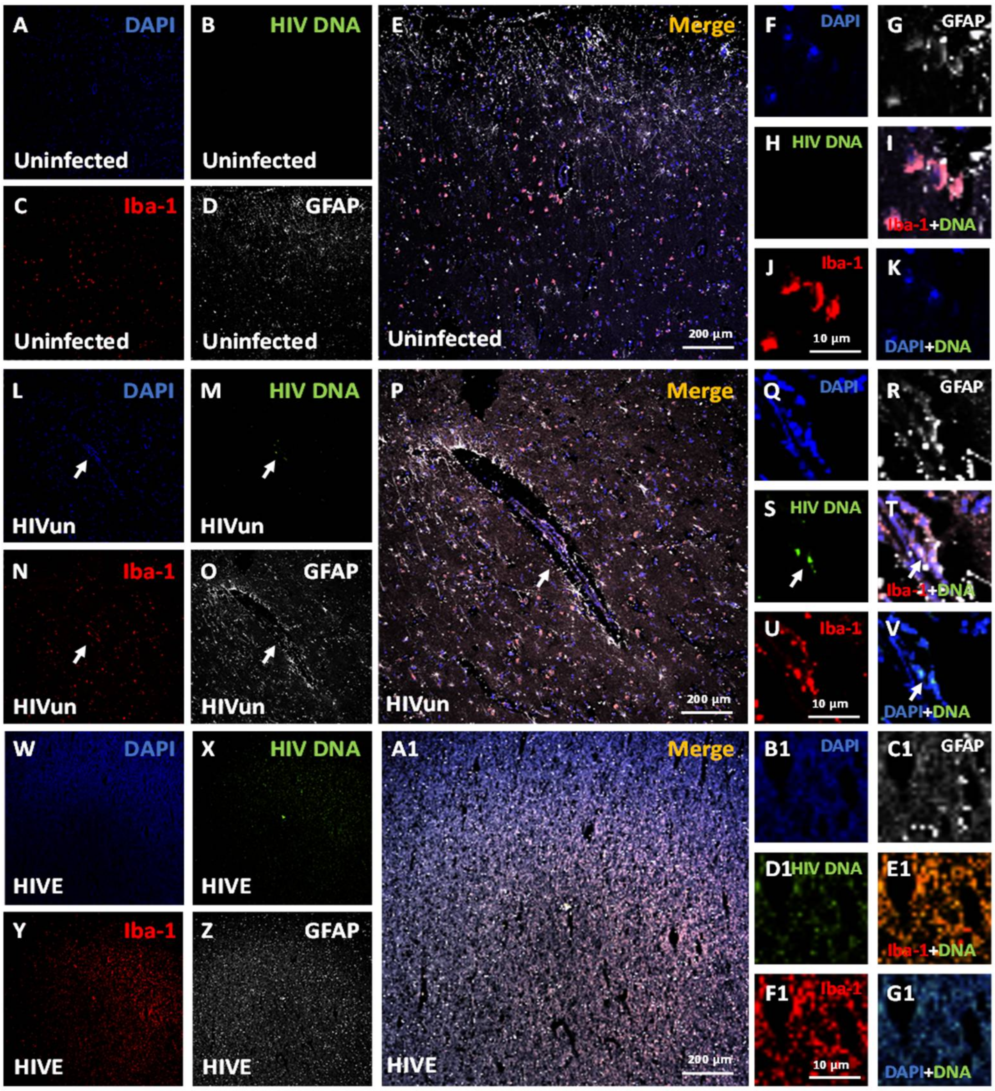

Supplemental Figure S1. Large tissue areas denote the rare distribution of viral reservoirs within the human brain of HIV-infected individuals with undetectable replication. (A-E) Representative large confocal images show the expression and distribution of nuclei (DAPI), HIV nef DNA (HIV DNA), macrophage/ microglia marker (Iba-1), astrocytes (GFAP), and merge of all colors in uninfected brain tissues (frontal cortex and hippocampus). (F-K) enlarged areas denote DAPI, GFAP, HIV DNA, Iba-1+DNA, Iba-1, and DAPI+DNA at large magnification in uninfected tissues. (L-P) Correspond to the same staining described above using HIV-infected tissues from individuals with undetected systemic replication (HIV_{un}). We must denote those large areas of the brain tissues analyzed were negative for HIV DNA. (Q-V) represent enlarged areas to denote DAPI, GFAP, HIV DNA, Iba-1+DNA, Iba-1, and DAPI+DNA at high magnification using HIV-infected tissues from individuals with undetected, low and high systemic viral replication. See the location of HIV DNA in the nucleus in viral reservoirs. (W-A1) Correspond to brain tissues obtained from HIV encephalitic tissues (HIVE) from early ART stages. Denote the high expression of Iba-1 and GFAP positive cells as well as the abundance of HIV DNA. (B1-G1) Corresponds to the high magnification of the areas described in the large magnification.

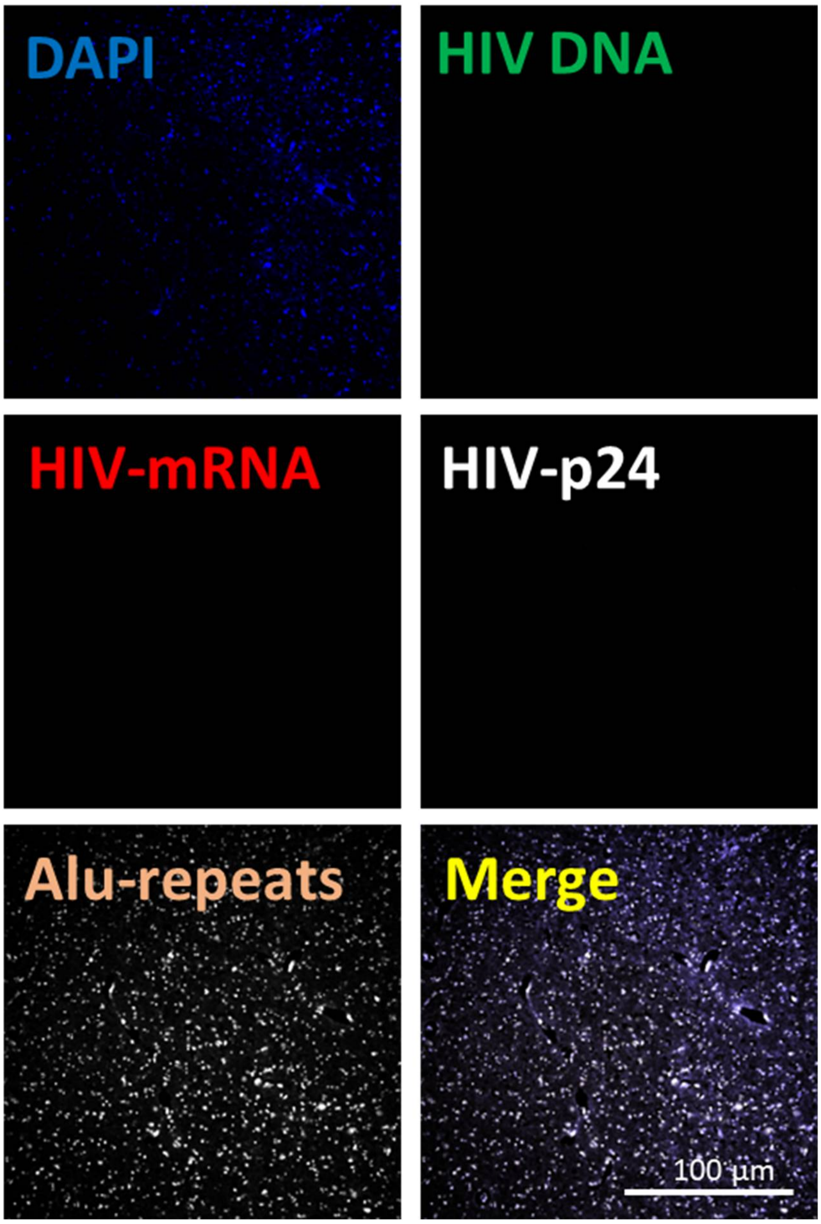

Supplemental Figure S2. Staining of human brain tissues obtained from uninfected individuals to determine the specificity of HIV DNA, mRNA, and viral HIV-p24 staining. Representative images of brain tissue sections from uninfected individuals stained for DAPI, HIV DNA, HIV-mRNA, HIV-p24 and Alu-repeats, and the merge of all colors. As expected, we only observed staining for DAPI and Alu-repeats in the uninfected brain tissues. There was no unspecific staining for HIV DNA, HIV-gag-pol mRNA, or HIV-p24 protein in uninfected tissues. Bar= 100 µm.

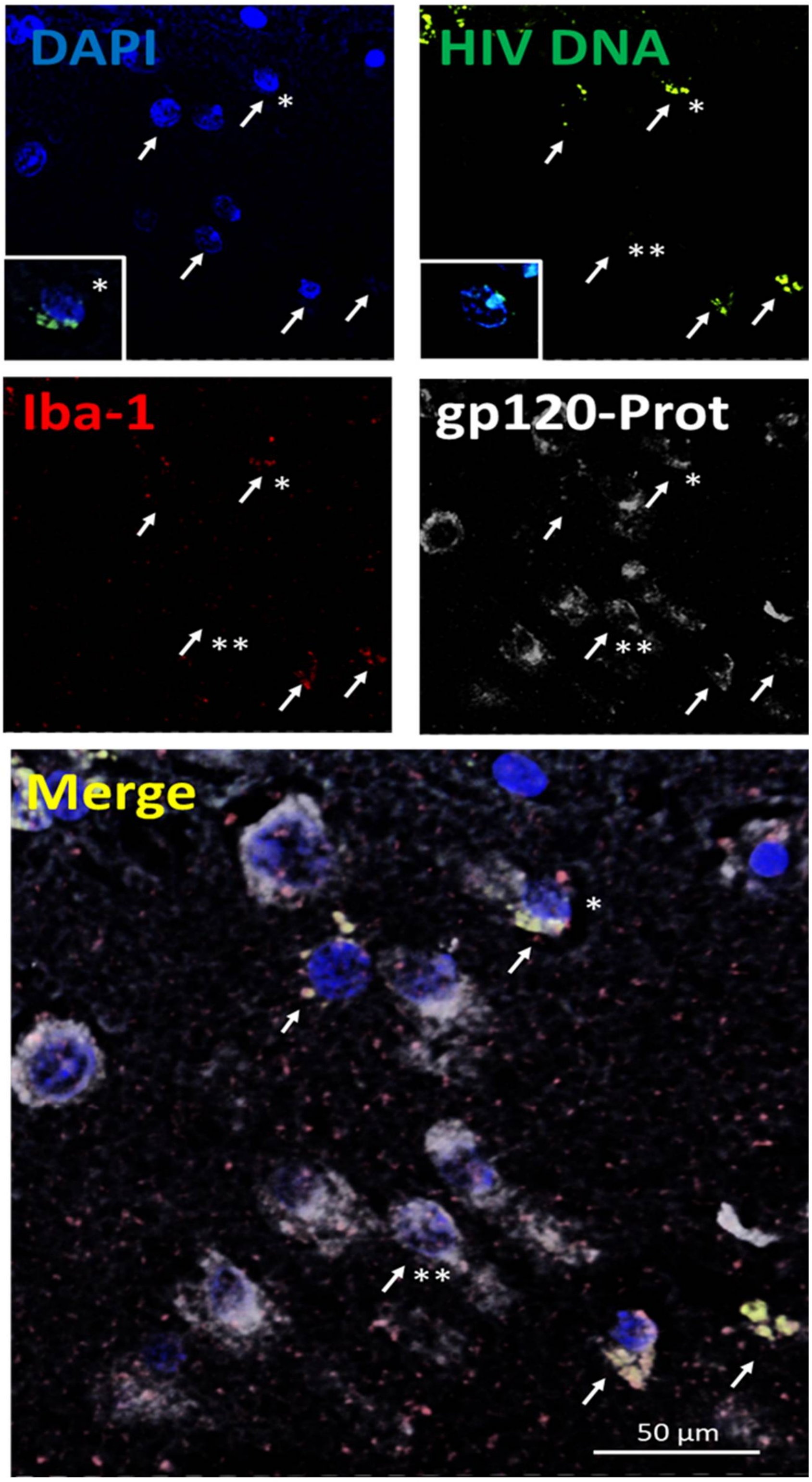

Supplemental Figure S3. High magnification of human brain tissue sections stained for HIV-gp120. Representative confocal images of brain tissue sections were obtained from individuals with undetectable or low systemic replication and stained for DAPI, HIV DNA, Iba-1, and HIV-gp120 protein. Arrows denote cells with HIV DNA signal in the nucleus, and asterisks represent cells with high gp120 accumulation and positive for HIV DNA within the nucleus. Bar= 50 µm.

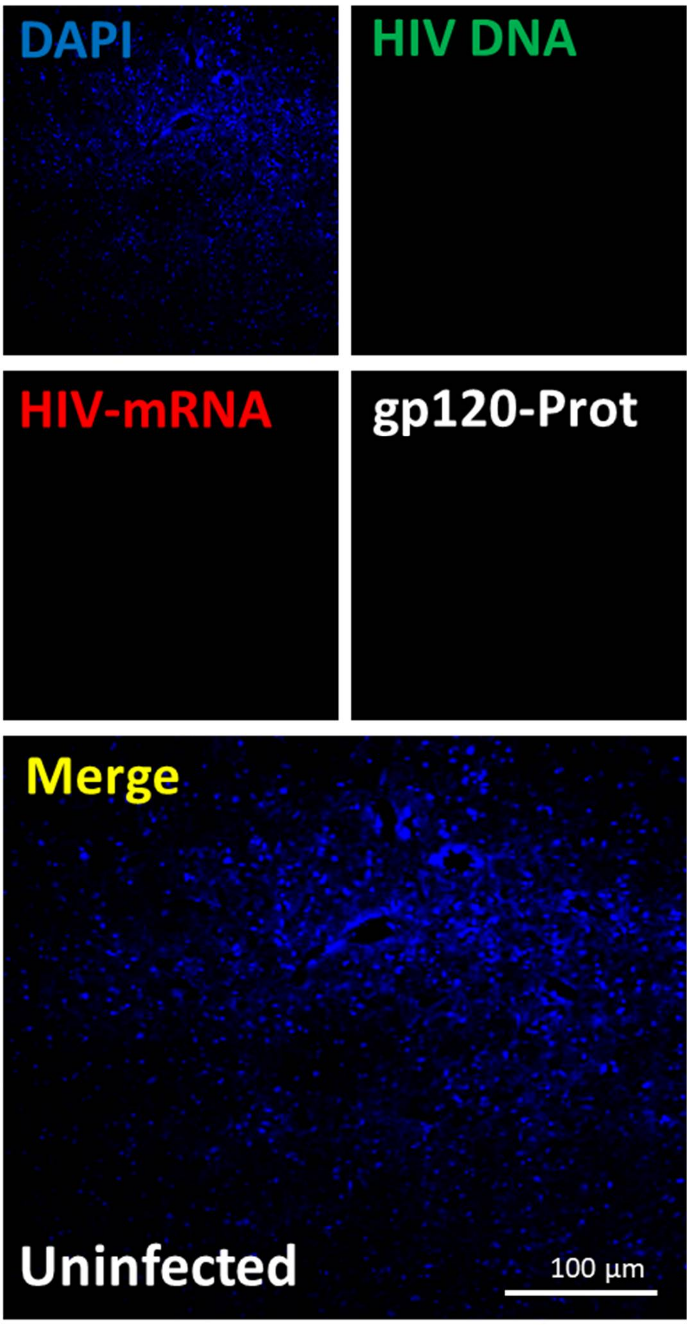

Supplemental Figure S4. Staining of human brain tissues from uninfected individuals to determine the specificity of HIV DNA, mRNA, and viral HIV-gp120 staining. Representative images of brain tissue sections from uninfected individuals stained for DAPI, HIV DNA, HIV-mRNA, and HIV-gp120, and the merge of all colors. In the uninfected brain tissues, we only observed staining for DAPI as expected. No unspecific staining for HIV DNA, HIV-gag-pol mRNA, or HIV-gp120 protein was detected in uninfected tissues. Bar= 100 µm.

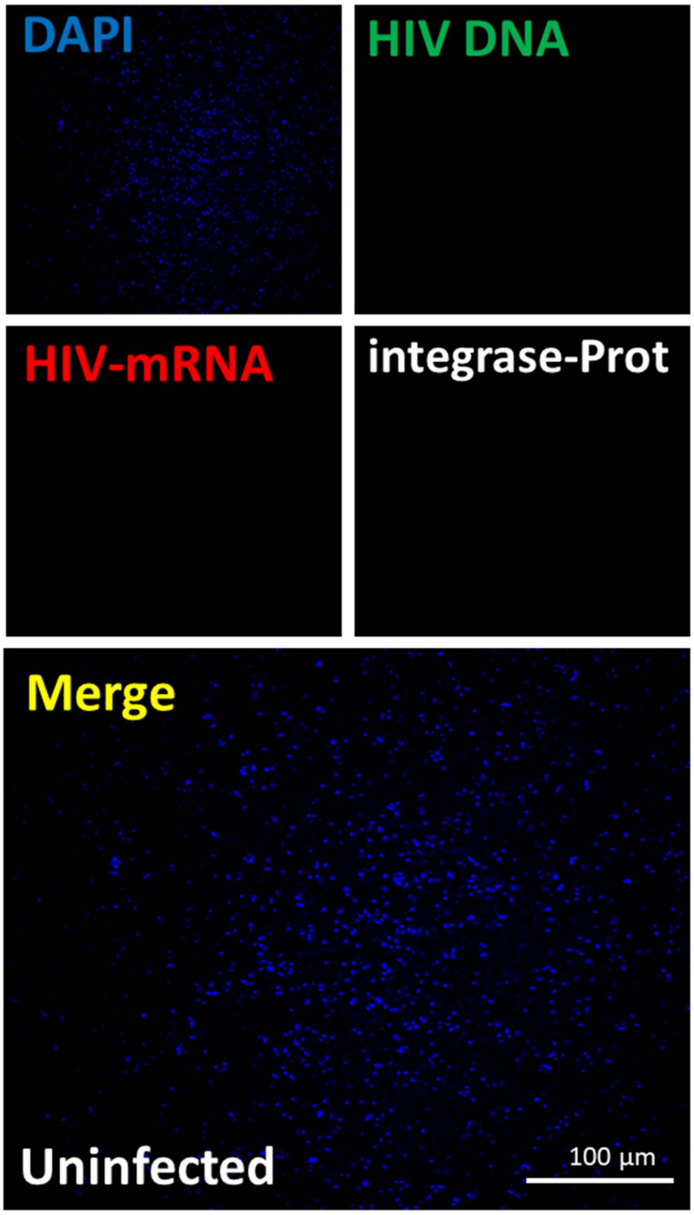

Supplemental Figure S5. Staining of human brain tissues from uninfected individuals to determine the specificity of HIV DNA, mRNA, and HIV-integrase staining. Representative images of brain tissue sections from uninfected individuals stained for DAPI, HIV DNA, HIV-mRNA, and HIV-integrase, and the merge of all colors. In the uninfected brain tissues, we only observed staining for DAPI as expected. There was no unspecific staining for HIV DNA, HIV-gag-pol mRNA, or HIV-integrase protein in uninfected tissues. Bar= 100 µm.

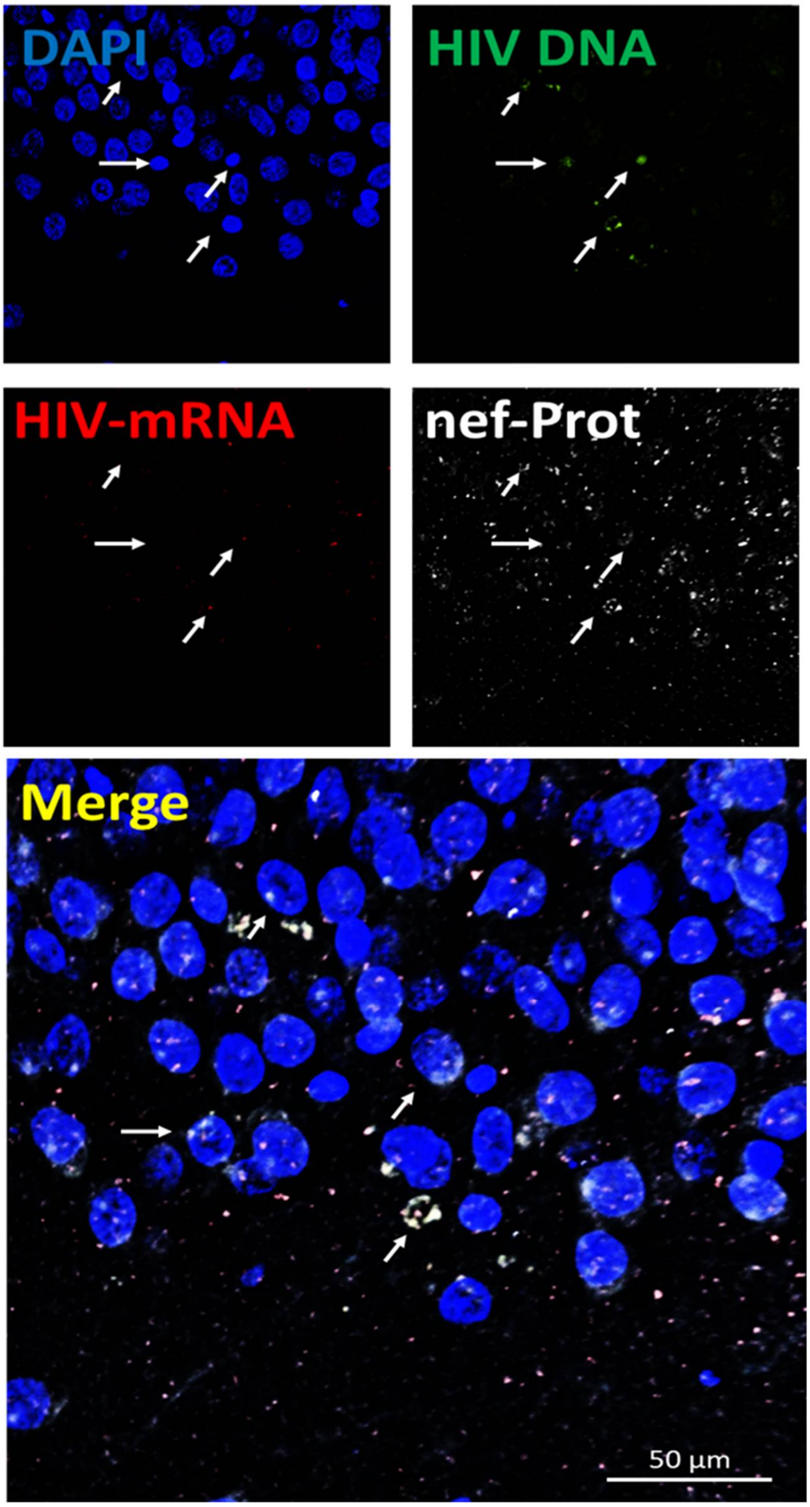

Supplemental Figure S6. High magnification of human brain tissue sections stained for HIV-nef protein. Representative confocal images of brain tissue sections were obtained from individuals with undetectable or low systemic replication and stained for DAPI, HIV DNA, Iba-1, and HIV-nef protein. Arrows denote cells with HIV DNA signals in the nucleus. Bar= 50 µm.

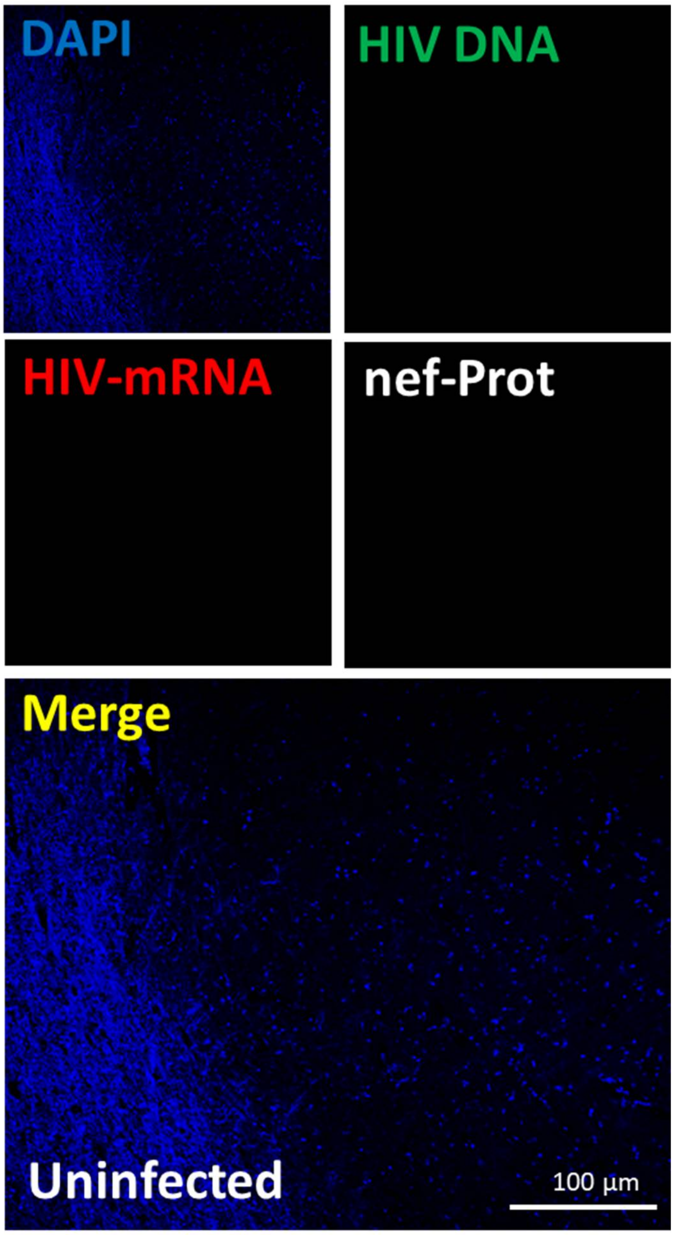

Supplemental Figure S7. Staining of human brain tissues from uninfected individuals to determine the specificity of HIV DNA, mRNA, and viral HIV-nef staining. Representative images of brain tissue sections from uninfected individuals stained for DAPI, HIV DNA, HIV-mRNA, and HIV-nef protein, and the merge of all colors. In the uninfected brain tissues, we only observed staining for DAPI as expected. No unspecific staining for HIV DNA, HIV-gag-pol mRNA, or HIV-nef protein was detected in uninfected tissues. Bar= 100 µm.

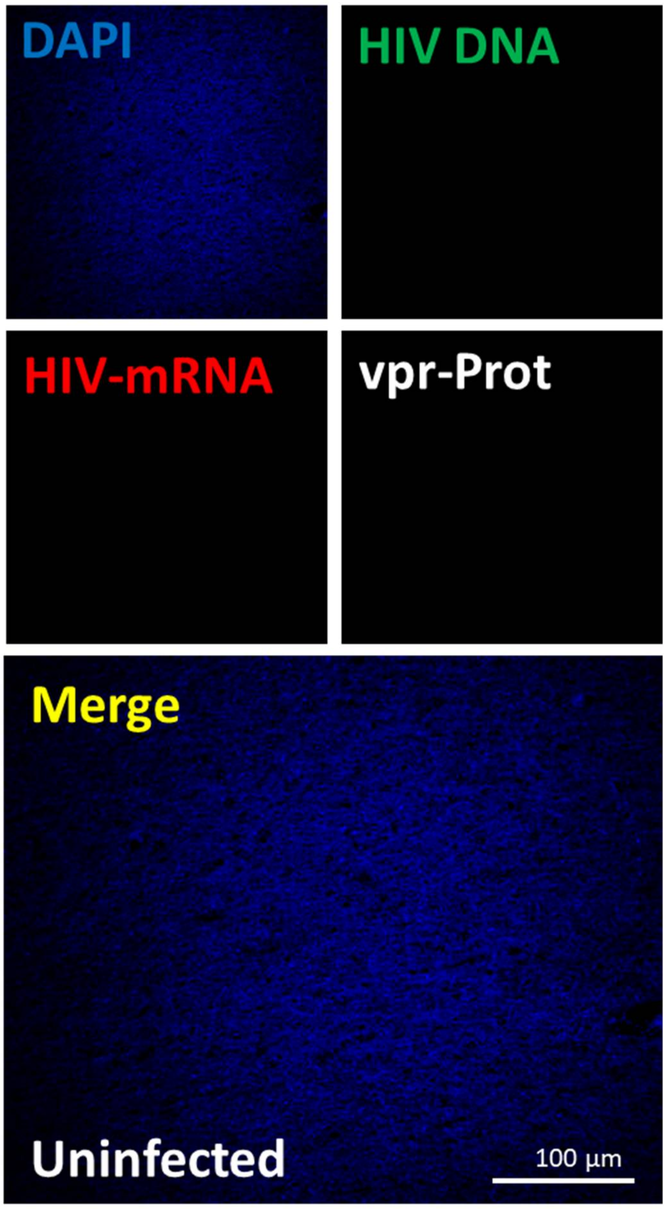

Supplemental Figure S8. Staining of human brain tissues from uninfected individuals to determine the specificity of HIV DNA, mRNA, and viral HIV-vpr staining. Representative images of brain tissue sections from uninfected individuals stained for DAPI, HIV DNA, HIV-mRNA, and HIV-vpr and the merge of all colors. In the uninfected brain tissues, we only observed staining for DAPI as expected. There was no unspecific staining for HIV DNA, HIV-gag-pol mRNA, or HIV-vpr protein in uninfected tissues. Bar= 100 µm.

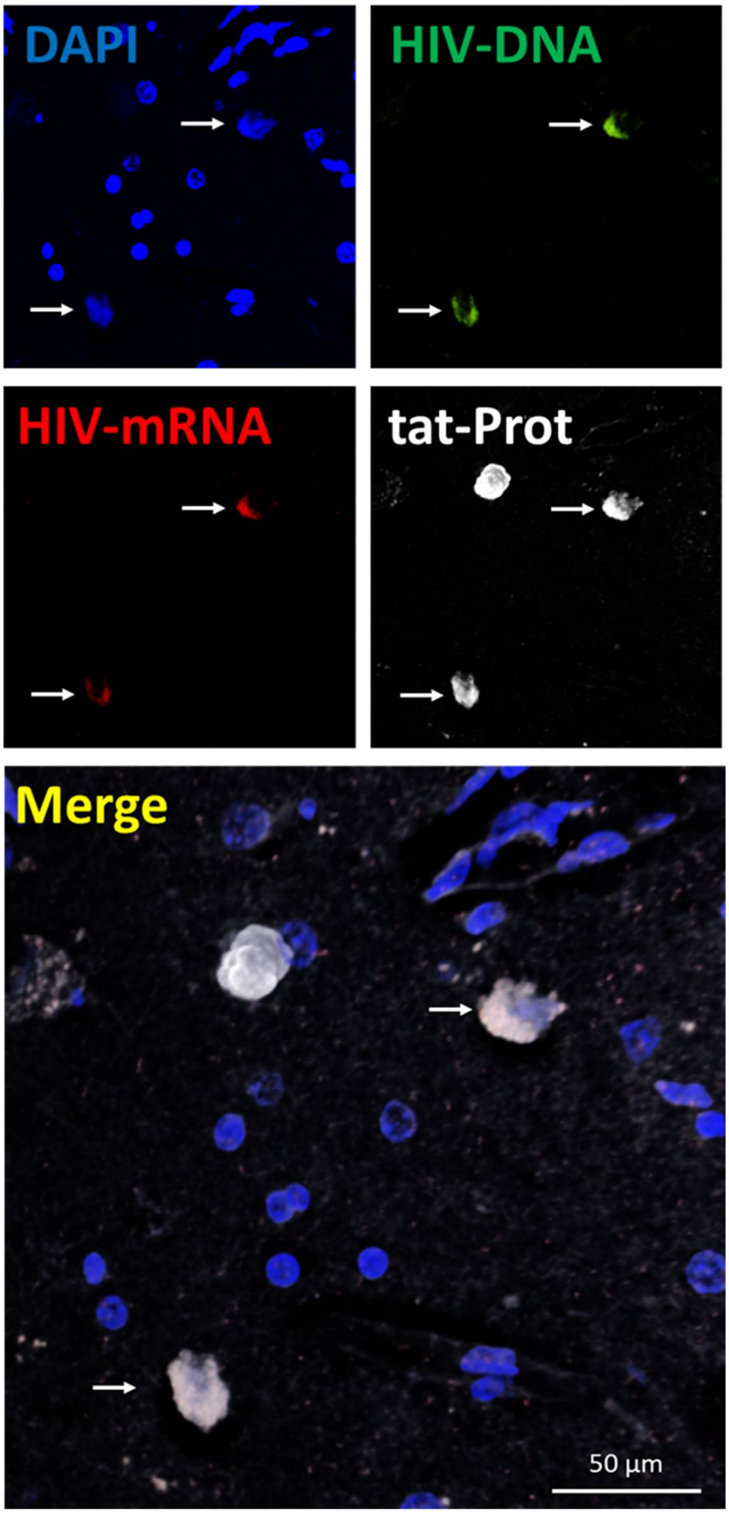

Supplemental Figure S9. High magnification of human brain tissue sections stained for HIV-tat. Representative confocal images of brain tissue sections were obtained from individuals with undetectable or low systemic replication and stained for DAPI, HIV DNA, Iba-1, and HIV-tat protein. Arrows denote cells with HIV DNA signal in the nucleus with high HIV-tat accumulation in the cytoplasm and nucleus. We also identified cells with high HIV-tat content but not HIV DNA signal in the nucleus. Bar= 50 µm.

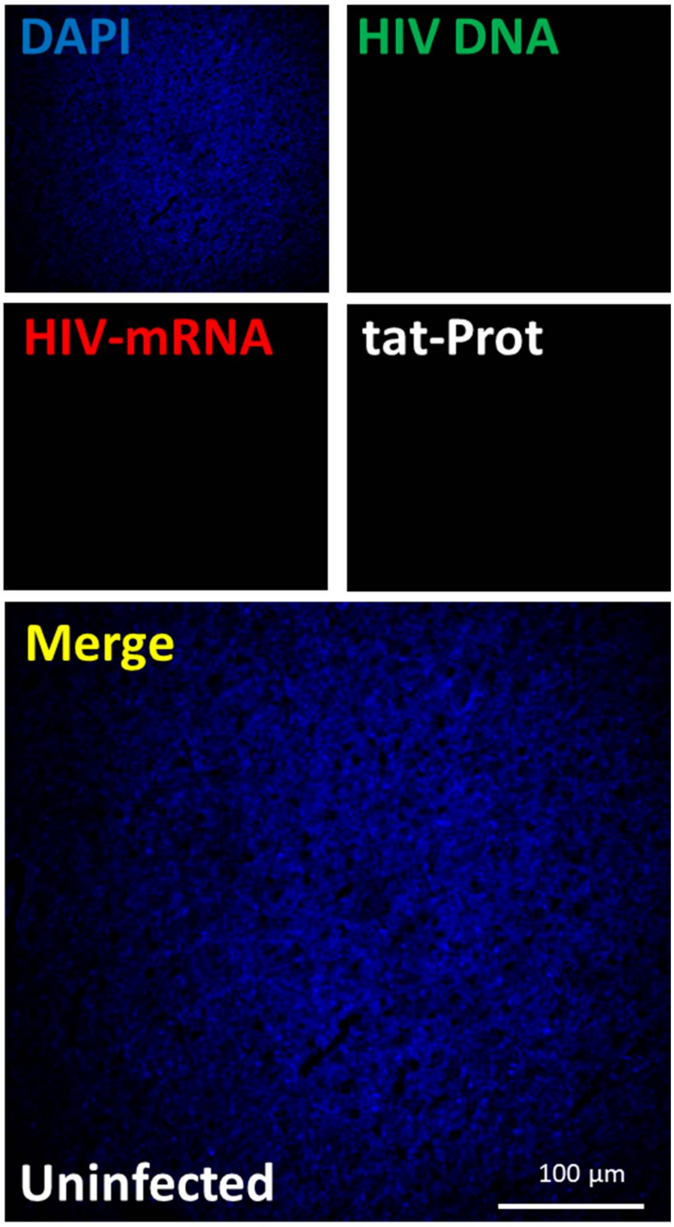

Supplemental Figure S10. Staining of human brain tissues obtained from uninfected individuals to determine the specificity of HIV DNA, mRNA, and viral HIV-tat staining. Representative images of brain tissue sections from uninfected individuals stained for DAPI, HIV DNA, HIV-mRNA, and HIV-tat and the merge of all colors. In the uninfected brain tissues, we only observed staining for DAPI as expected. There was no unspecific staining for HIV DNA, HIV-gag-pol mRNA, or HIV-tat protein in uninfected tissues. Bar= 100 µm.

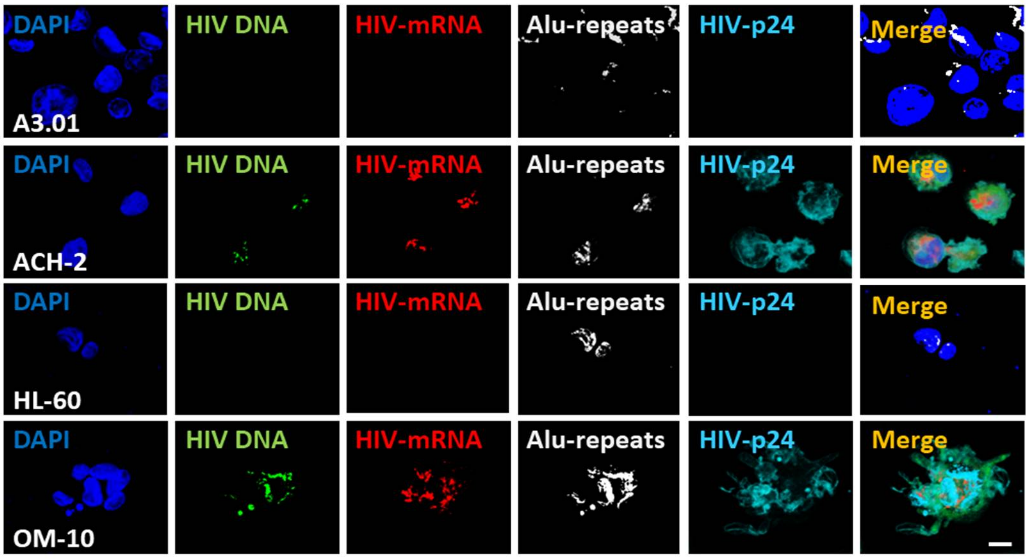

Supplemental Figure S11: Colocalization examples of DAPI, HIV DNA, HIV-mRNA, Alu-repeats, and HIV-p24 in A3.01, ACH-2, HL-60, and OM-10 cells. Representatives images show uninfected cells (HL-60 and A3.01) and HIV-infected cells (ACH-2 and OM-10) for distribution, colocalization, and sensitivity evaluation. ACH-2 and OM-10 contain only one copy of HIV-integrated DNA. HIV-nef DNA probe, HIV-gag-pol mRNA probe, and HIV-p24 protein were detected in infected cells and not in uninfected cells. DAPI and Alu-repeats DNA probes are shown as controls. Bar= 20 μm.

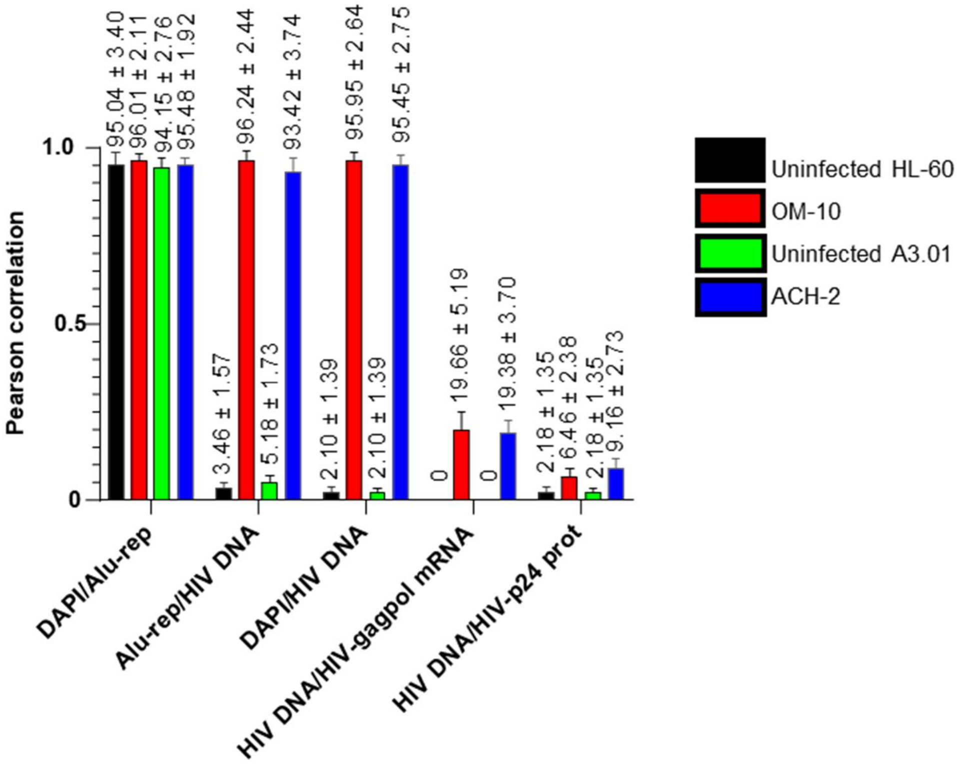

Supplemental Figure S12. Pearson's colocalization coefficient among the different HIV and host markers was examined. Analysis of the confocal images to determine the degree of colocalization of the host (Alu and DAPI) and viral (HIV DNA, viral mRNA, and proteins) markers using uninfected cells (HL-60 and A3.01) and HIV-infected cells (ACH-2 and OM-10). The analysis was performed to compare the colocalization of positive pixels for each marker using NIS Elements. Percentages obtained for each type of cell are described.
